# Risk Management in the New Frontier of Professional Liability for Nosocomial Infection: Review of the Literature on Mycobacterium Chimaera

**DOI:** 10.3390/ijerph17197328

**Published:** 2020-10-07

**Authors:** Matteo Bolcato, Daniele Rodriguez, Anna Aprile

**Affiliations:** Legal Medicine, Department of Molecular Medicine, University of Padua, Via G. Falloppio 50, 35121 Padua, Italy; danielec.rodriguez@gmail.com (D.R.); anna.aprile@unipd.it (A.A.)

**Keywords:** Mycobacterium chimaera, medico legal assessment, professional liability, informed consent, clinical risk management

## Abstract

*Background*: *Mycobacterium chimaera* (MC) is of recent origin and belongs to the large family of non-tuberculous mycobacteria. In recent years, it has shown a high infectious capacity via the aerosol produced by operating room equipment, such as heater–cooler units (HCU). The infection has a long latent period and high mortality rate. Genetic and epidemiological studies have shown that there is a clear link between the infection and a specific HCU model manufactured by LivaNova/Sorin. There is, therefore, a strong possibility that contamination occurs during device construction. The objective of this article is to describe the characteristics of this particular infection in view of the medico–legal implications on professional liability, specifically focusing on current evidence regarding contamination prevention. *Methods*: we have analyzed the clinical characteristics and data from the autopsic investigations performed on a patient who died as a result of MC infection, in addition to analyzing all pertinent recommendation documents available internationally. We searched for all articles in the literature available on MEDLINE between 1995 and 30 July 2020, using the search words “Mycobacterium chimaera”. We then analyzed those articles and reported only those that provide useful information regarding prevention techniques for containing dissemination and contamination. *Results*: the literature review produced 169 results that highlight the need to develop systems to mitigate and eliminate the risk of MC infection in operating rooms such as physical containment measures, e.g. device replacement, use of safe water, providing patients with information, and training healthcare professionals. *Conclusions*: from a medico–legal viewpoint, this particular situation represents a new frontier of professional liability, which includes manufacturers of electromedical equipment. In order to comprehend the true extent of this silent global epidemic, the development of an organic, preventative monitoring system is essential.

## 1. Introduction

*Mycobacterium chimaera* (MC) belongs to the large family of non-tuberculous mycobacteria (NTM), which are commonly found in the environment, especially in water. This particular type of mycobacterium was only identified in 2004, when Tortoli et al [1] carried out molecular tests and identified particularly aggressive, epidemiological characteristics that led to the proposal of a new taxonomic classification, *Mycobacterium chimaera*. The term *chimaera* is used due to its hybrid characteristics as compared with the more widely known mycobacteria [2,3] and similarity to *M. avium* and *M. intracellulare*; the name refers to the creature described in Greek mythology composed of the parts of different animals.

In subsequent years, references to MC in the literature from many parts of the world have increased; the mycobacterium has often been found in patients affected by respiratory problems among others, indicating rising dissemination on a global level [4,5,6,7,8,9,10,11,12].

The clinical manifestations of an MC infection can be varied and may include systemic and aspecific signs and symptoms, such as fever, asthenia, easy fatigue, and other more specific symptoms, such as the formation of emboli on cardiac valves or structures involved in surgery. These formations can then embolize and cause neurological, ocular and auditory damage [13,14]. The infection has a long latent period that can extend even beyond 70 months. On average, patients present with no symptoms until approximately 20 months [15,16], which makes diagnosis complex. To date, treatment with antimycobacterial drugs [17,18,19] is still uncertain and the mortality rate remains above 50% [20,21,22].

Although MC in water [23] is generally not dangerous to immunocompetent humans, since 2014 it has been indicated as a cause of infection in patients who have undergone heart surgery with exposure to contaminated heater–cooler units (HCU) [24]. Several patients have been affected by MC with a long incubation period following cardio-surgical operations, despite the small surgical opening in the chest [25,26,27,28,29,30].

Epidemiological studies have demonstrated a clear link between the infection and a specific HCU model: the 3T device manufactured by LivaNova/Sorin. In particular, the tests conducted at the manufacturing site revealed that the HCU water tanks and water present in the system pump assembly area were contaminated. Genetic investigations on the strains of MC on environmental and clinical isolates from patients from three different European countries showed almost identical genome sequences [31]. One study on the whole genome sequence of clinical isolates from patients infected with MC who had undergone open-heart surgery in hospitals in the USA showed few single nucleotide polymorphism differences [32]. Apart from this, there are no references in the literature to cases of infection associated with HCU devices produced by manufacturers other than LivaNova. These factors show the high probability that contamination occurs at the manufacturing site before the devices are dispatched to hospitals. The characteristics of MC enable it to lodge on the device and form a biofilm that is particularly resistant to the disinfection protocols in place. The assumption that contamination first begins in the production stage is supported by the fact that no other operating room equipment such as extracorporeal membrane oxygenation (ECMO) devices, though considered a potential source of infection, has been shown to have caused a patient to contract MC, and none of the studies has detected any contamination of the air in the environment [33]. This confirms the hypothesis that HCU contamination occurs during the manufacturing process.

In order to comprehend the infection mechanism, several studies have been conducted, the results of which show that the exhaust vent from contaminated HCUs can transmit contaminated aerosol to the surgical field by laminar airflow [34]. It has been demonstrated that the probability of NTM infection increases in proportion to the time the patient spends connected to a running HCU; surgeries that exceed 5 hours present a significant risk [35]. These findings suggest that the source of infection is, therefore, the contaminated aerosol that, by means of insufflation, comes into contact with parts of the body exposed during surgery and that MC is able to lodge and create a thin biofilm on body structures, particularly cardiac structures, and manifest over long periods of time [36]. The objective of this article is twofold: (1) to describe the specific characteristics of this infection from a clinical and forensic point of view; (2) to analyze the correlated medico–legal implications on professional liability, specifically focusing on current evidence regarding contamination prevention.

## 2. Materials and Methods

In order to achieve the objectives of this study, we have used various sources of information: (1) a clinical and autoptic case study which demonstrates the characteristics of the infection from a forensic point of view. The current literature contains no case studies, which describe the forensic pathology aspects of this disease; (2) official documents issued by international institutions to contain the infection in various parts of the world; (3) a review of the literature concentrating on aspects pertaining to current evidence regarding prevention.

### 2.1. Case Report

We have analyzed the clinical characteristics and data from the autopsic investigations performed on a patient who died as a result of MC infection.

### 2.2. Official Recommendation Documents 

We analyzed the official documents available on an international level and the indications provided by the Italian Ministry of Health and arranged them in chronological order so as to be able to evaluate not only the current but also the previous state of the evidence. The results may prove to be particularly useful in medico–legal court proceedings.

### 2.3. Literature Review

We ran a search for all articles in the literature available on MEDLINE between 1995 and 30 July 2020, using the search words “*Mycobacterium chimaera*” with the objective of cataloguing articles and documents that contain useful information on prevention techniques for containing dissemination and contamination within the broader program of clinical risk management. All sources have been evaluated independently by the authors to determine their relevance to the present study and then selected for inclusion by all the authors.

## 3. Results

### 3.1. Case Report

**Figure 1:**
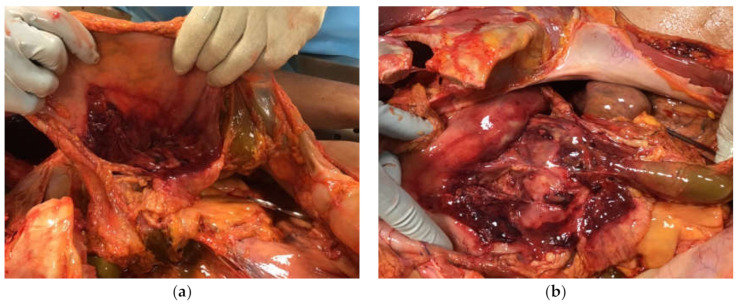
(**a**,**b**) Diffuse abdominal inflammation due to pancreatitis.

**Figure 2:**
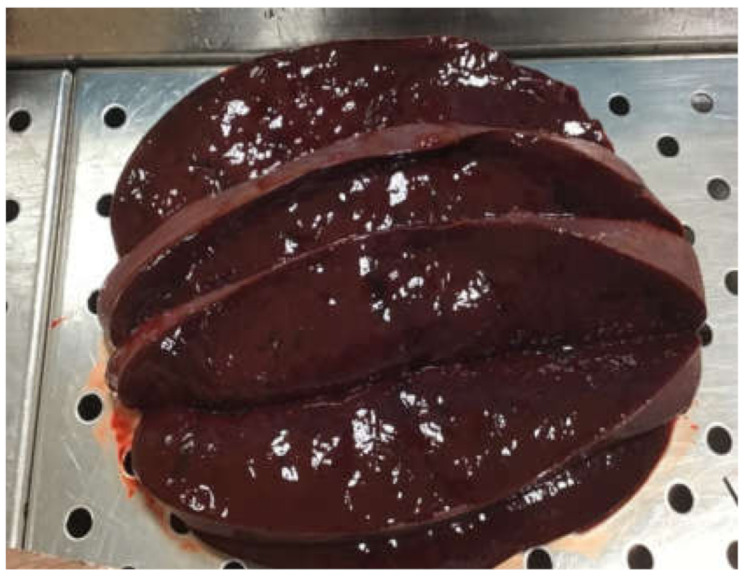
Spleen characterized by splenomegaly.

**Figure 3:**
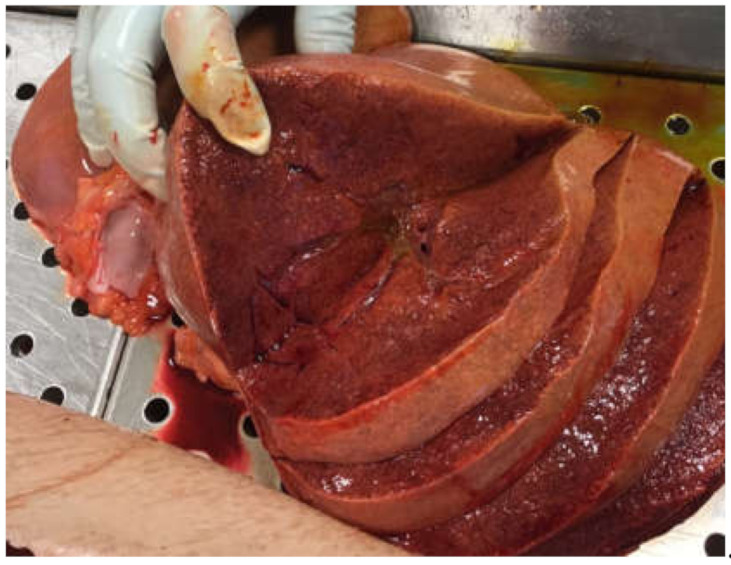
Cirrhotic degeneration of the liver.

In 2015, a 50-year-old man underwent surgery due to mitral valve insufficiency. The intra- and post-operative period appeared regular with good recovery. After approximately 12 months, the patient noted general symptoms of fever and fatigue, and valvular endocarditis caused by MC was detected, which subsequently required replacement surgery. Antimycobacterial therapy was administered, first with rifampicin, ethambutol, and clofazimine, and then continued with ethambutol and clofazimine. For several years, the infection seemed to be under control, although the patient did show inability to exert himself, marked asthenia, and susceptibility to infections. In 2020, five years after surgery, the patient’s condition rapidly deteriorated due to weight loss, worsened asthenia and skin discoloration. His hematochemical tests highlighted significant renal and hepatic insufficiency with elevated pancreatic indices. Moreover, 30 days after the onset of these symptoms, the patient was hospitalized due to severe pancreatitis and died a few days later. The autopsy revealed the presence of pancreatitis involving the omental tissue (Figure 1a,b), a significant increase in the dimension (approximately 30 × 15 cm) and weight (1.3 kg) of the spleen (Figure 2), congestion, and cirrhotic degeneration of the liver (Figure 3), in addition to severe destruction of the renal parenchyma.

### 3.2. Review of Official Recommendation Documents

The analysis of official recommendation or communication documents from the designated agencies showed that the issue of MC infections associated with HCU devices was brought to the attention of the international community and supervisory boards in 2015 when the European Centre for Disease Prevention and Control (ECDC) published its first Rapid Risk Assessment [37] document and subsequent updates [38]. These documents proposed measures to reduce the risk of infectious transmission through the use of HCU devices and the identification of cases of infection. A dedicated task force, composed of a group of supervisory experts from the European Commission, was set up to analyze this issue and collaborate with member states to identify the most appropriate corrective measures. Centers for Disease Control (CDC) in the USA [39] and Australia [40] took similar precautions.

In Italy, on 9 January 2019, the Italian Ministry of Health issued recommendations for controlling MC infections. These recommendations designated the National Institute of Infectious Diseases (INMI) “L. Spallanzani” as the national reference laboratory for confirmation of cases, molecular diagnosis of MC isolates, data collection related to molecular diagnoses carried out in other laboratories and conservation of bacterial strains.

For the Italian field, the manufacturing company has issued various safety notices and recommendations for healthcare operators on correct cleaning and disinfection procedures.

The first notice sent to healthcare facilities that had been supplied with the aforementioned HCU devices and others that may potentially be at risk and published on the Italian Ministry of Health’s website dates back to 6 February 2015. It reported the risk of contamination of the surgical field from the aerosol produced by HCU devices and the need to take urgent action to disinfect, test the water used, and collect microbiological samples.

Table 1 lists all the notices issued by the manufacturer on this topic that are present on the Ministry’s website. Starting from 11 November 2016 [41], it was clearly recommended that:

(a) HCU devices, where contamination by MC is suspected or certain, must be removed from the operating room or from use as soon as possible.
(b) Facilities equipped with devices where no MC contamination has been confirmed must:
  1. Observe the instructions for use for heater–cooler devices, particularly as regards cleaning and disinfection.
  2. If the operating room available permits, direct or channel the heater–cooler’s exhaust vent away from the patient, e.g. towards the operating room exhaust vent in line with the safety notice on the “Risks of Mycobacteria Infection in Cardiac Surgery”.
  3. Monitor the water quality in accord with the safety notice on the “Risks of Mycobacteria Infection in Cardiac Surgery”.
  4. Use new accessories, tubing, and connectors to prevent recontamination if using a different heater–cooler device.

In line with these notices, the Veneto regional authority issued Regional Government Decree No. 999, 12 July 2019 (operating guidelines for supervising and controlling mycobacterium chimaera infections associated with surgical operations using heater–cooler units), urging hospitals on a local level to comply with the direction and compile a report to be sent to the Regional Government detailing how individual hospitals have implemented these guidelines.

Furthermore, the abovementioned decree requires that doctors immediately report cases of MC infection, even if only suspected, in patients who have undergone extracorporeal circulation using an HCU to the Medical Facility Directorate, using the designated form. The Directorate will in turn forward that report to the competent Prevention Department and the Regional Department named “Azienda Zero”. The form should be submitted along with a case report describing the prevention and control protocols in place locally. This should be updated as further information becomes available. In addition, the document designates the Department of Microbiology and Virology of the Hospital of Padova as the regional reference laboratory for MC infections, to which isolates from potentially infected patients should be dispatched. 

### 3.3. Literature Review

**Figure 4:**
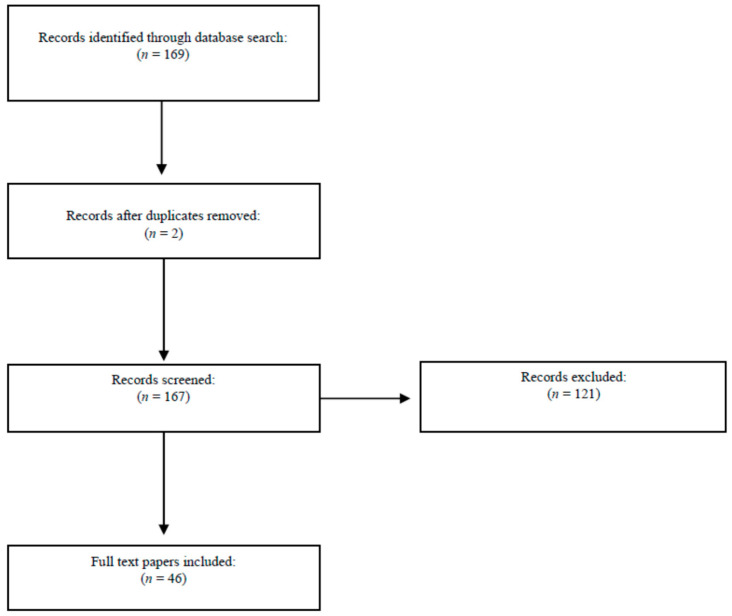
Flowchart depicting the choice of studies.

The MEDLINE search produced 169 results (Figure 4). Although the first article to mention MC dates back to 2004, our review shows that only since 2016 has significant interest been manifested in the topic, which has increased over the last four years. The search yielded 36 articles from the last year alone. The literature also showed one systematic, bibliometric analysis [52], and 20 literature review-type articles. However, no randomized controlled trials or meta-analyses are present. The vast majority of the articles deal with the topic from a microbiological, infectivological and, more recently, genetic viewpoint. Only one article examines the topic from a medico–legal viewpoint [53].

In addition, the review highlighted the need to develop systems to mitigate and eliminate the risk of MC infection inside operating rooms [54].

#### 3.3.1. Physical Containment Measures

The scientific literature reviewed [55,56] coheres with the indications provided by the aforementioned international supervisory boards who, in line with the current evidence, recommend the implementation of the following physical containment measures:

(a) Position the HCU outside the operating room, connected through a wall port; thus, ensuring positive pressure inside the operating room.

Based on current understanding, this is the safest approach considering the fact that transmission via aerosol from the device has been verified. However, physically moving the device may involve significant structural and technical adjustments to the healthcare facilities, which will inevitably affect operational activities in and around the operating room [57].

Non-structural solutions, such as transferring the device to an adjacent room, leaving the door ajar for the tubing, do not seem to be sufficient to prevent aerosol circulation [58]. Directing the device’s exhaust vent away from the patient does not in itself guarantee protection from the risk of infection; in addition, any structural modification of the device must be approved by the manufacturer.

(b) Replace all Stöckert 3T HCU devices manufactured by LivaNova prior to September 2014.

Though recommended, this solution does not guarantee protection from the risk of infection in and of itself given that contamination could have occurred post September 2014. Nevertheless, devices antecedent of that period are still considered at greater risk [59].

(c) Implement water/air monitoring and testing systems.

MC and other mycobacteria have been detected in the water from mains water pipes; their presence and persistence is typical of these organisms, which are particularly resistant to common disinfectants [59,60]. Physical filters on tap water can be used, although one study [61] shows that few have significantly reduced the transfer of mycobacteria. However, though these filters may be useful in the general domestic and hospital context, they are unlikely to guarantee the complete safety of the tap water used in an HCU device.

Therefore, it is recommended that only sterilized or filtered water, uncontaminated by mains water, be used, provided the device has not been contaminated previously.

Regarding periodical testing programs on the water and air produced by the devices, opinions in the literature differ. While potentially useful, such routine testing is not yet recommended because the results have not always been completely comparable and reliable; they can therefore be falsely reassuring [62]. To date, there is no standard procedure for collecting and sampling water and air isolates; moreover, several weeks are required to obtain results. This may create uncertainty as to proper device maintenance, so much so that the Canadian authorities have advised against routine testing [63].

#### 3.3.2. Operator Training and Monitoring Patients at Risk

MC infections show an often symptomless latent period that can be considerably lengthy, which renders identification and careful, preventative monitoring of at-risk patients complex, especially for the purposes of recognizing the infection early and treating it with all therapeutic options available.

To that end, in line with the Italian Ministry of Health’s recommendations on controlling MC infections dated 9 January 2019, cardiologists and cardiac surgeons who perform follow-ups on cardiac surgery patients, in addition to doctors of general medicine, must receive proper training and information on the risks associated with post-cardiac surgery MC infections, common signs and symptoms, diagnostic tests, and reporting methods.

Despite the difficulty in identifying cases by means of retrospective investigations, given the long latent period and often aspecific symptoms, the 9 January 2019 recommendation document initiated a retrospective analysis program on a regional level using information acquired from tests performed at microbiology laboratories and hospital discharge forms.

Concerning the former, laboratories are requested to produce a list of all MC isolates processed and verify whether those patients had undergone cardiac surgery with the use of an HCU device, and, if affirmative, review the clinical records and insert those patients into a monitoring program.

As regards the latter, hospital discharge databases make it possible to find all patients who have undergone at-risk cardiac surgery, especially cardiac valve surgery, and to review the clinical records in order to identify patients whose clinical and laboratory results are compatible with MC infection. If identified as positive, the search can be extended to include other cardiac surgeries performed in the same facility in the same time period that were initially considered lower risk.

Both approaches facilitate the identification of cases of infection and monitoring their development for clinical and epidemiological purposes in order to comprehend the properties of this slow global epidemic, and for research purposes regarding the particular infectious agent.

In the Veneto region, on receipt of the aforementioned national indications, an information campaign was initiated for the benefit of patients who between 2010 and 31 December 2017, underwent cardiac surgery with extracorporeal circulation, and who are at risk of invasive infection by MC. In addition, the local healthcare authorities sent a specific information pamphlet to patients and doctors of general medicine. These information pamphlets factor in an extended incubation period of up to 6 years.

#### 3.3.3. Information to Provide to Patients

Information and consent are fundamental elements of the doctor–patient relationship, underpinned by recognized legal and cultural foundations [64,65]. In regards to the risk of MC infection, activities designed to monitor and identify those at risk are essential elements in ensuring the right to information of patients who have or might have undergone at-risk procedures. There is a more complex issue that concerns patients undergoing cardiosurgery procedures today with the use of potentially at-risk HCUs. The literature [66] suggests that until effective risk mitigation measures can be put in place, patients must be provided with information on the potential risk of exposure before proceeding with the operation. This suggestion is appropriate as the patient must possess complete information on the associated risks in order to make a decision. Nonetheless, it is clear that providing the patient with adequate information regarding risks, such as MC infection, does not constitute grounds for excluding potential professional liability in the event of a nosocomial infection contracted during surgery. Italian law stipulates that patient safety must be guaranteed while undergoing necessary treatment; tolerating the presence of a risk that has been objectified and reported on for some time now is not acceptable.

## 4. Discussion

The dissemination and danger of infections contracted in hospital settings represent a serious public health and medico–legal problem [67,68,69]. In recent decades, there has been an increase in awareness, thereof, due to the social and patient safety implications, an issue that, in Italy, was highlighted by the recent law on the safety of care. This law, no. 24/2017 [70,71], set forth the need to implement clinical risk management programs in all hospital settings to prevent the occurrence of such infections [72,73].

Cases of MC infection are particularly difficult and complex for various reasons: a) the time between infection and manifestation of symptoms is extremely long, and the clinical characteristics can vary greatly and are not yet fully understood and described in the literature; b) the characteristics of this particular mycobacterium, the route of infection and the symptomatological presentation are not widely recognized by doctors, who therefore tend not to suspect infection; c) many diagnostic tests for MC are slow and of low sensitivity; a DNA test is needed to confirm identification; d) the hospital infection route is very specific and occurs through routine processes employed within operating rooms; e) elimination of MC biofilms from surfaces by means of routine disinfection procedures poses significant difficulties.

The study of this specific mycobacterium is complex and further microbiological studies are required to be able to fully identify the transmission characteristics thereof and to measure the efficacy of the prevention measures that have been introduced. For the purposes of risk containment, the evidence considered highlights the need to monitor obsolete devices and arrange for replacements, along with the need to position the device outside the operating room using methods to prevent potentially contaminated aerosol from reaching the patient. In addition to these precautions, the use of safe water only in conjunction with uncontaminated devices can protect the patient. However, there is no significant evidence to warrant routine water and air sampling.

From a training perspective, much can be done. Training for all personnel actively involved in operating rooms is essential for ensuring maximum patient safety. Training healthcare professionals involves providing information, on the methods and presentation of MC infections, to all who work in cardiology and cardiac surgery departments, including cardiovascular perfusion technicians, radiology nurses, and technicians practicing in that department, in addition to primary healthcare and internal medicine physicians.

In order to comprehend the true extent of this silent global epidemic, it is essential to develop an organic preventative monitoring system for at-risk patients undergoing cardiac surgery and to conduct retrospective analyses in order to monitor closely patients who have been exposed to the risk of infection.

Identification of cases of infection cannot be left entirely to the ability of doctors; however, comprehensively and thoroughly trained and informed regarding MC, to arrive at a diagnosis from the symptomatology of an individual patient via laboratory tests. MC is an insidious bacterium because it has an aspecific symptomatology, an extremely late manifestation in relation to contact with the host in addition to a completely asymptomatic latent period. An isolated diagnosis of an individual case by an individual doctor is uncertain and, in any case, late as regards the probability of therapeutic success in the specific patient, the identification of the risk and the consequent prevention of other cases originating from the same source.

Since there is increasing evidence of the possibility of HCU contamination from various non-tuberculous mycobacteria, and other opportunistic pathogens and consequent dissemination via aerosol, it is appropriate that monitoring not be limited to MC infection, but extended to all these additional agents.

The general objective of such monitoring is to make a global contribution to the identification of infectious risks connected to the use of electromedical devices in cardiac surgery.

## 5. Conclusions

From a medico–legal point of view, MC infection represents a new frontier of hospital infection liability in that, not only are the preventative and diagnostic activities of doctors implicated, but also those of the manufacturer. Given this potential twofold liability, litigation involving MC infections requires careful medico–legal analysis, including an evaluation of the quality and timeliness of measures the specific hospital has put in place in view of the recommendations issued by the supervisory boards. It would also be necessary to evaluate on a case-by-case basis whether the manufacturers and Ministry of Health’s recommendations, if applied, were sufficient to counteract the infection effectively and reliably.

New knowledge on the topic of infections associated with electromedical apparatus has clear repercussions on both clinical risk management in regards to risk identification and elimination, and on medico–legal evaluations on healthcare liability, particularly in the context of reconstructing the causal link of infections with long incubation periods and modest clinical manifestations.

## Figures and Tables

**Table 1:** List of safety notices issued in Italy on the dissemination of MC by HCUs.

Safety Notice: 6 February 2015. Disinfection and cleaning of Sorin heat generators [42]
Safety Notice: 9 December 2015. Revised washing and decontamination procedures [43]
Safety Notice: 15 June 2016. Recall on disinfection and cleaning of oxygenator mounting brackets [44]
Safety Notice: 1 July 2016. Recall on disinfection and cleaning of oxygenator mounting brackets [45]
Safety Notice: 1 July 2016 (2nd) Disinfection and cleaning of pediatric oxygenator mounting brackets [46]
Safety Notice: 8 July 2016. Safety information on heat generator system [47]
Safety Notice: 11 November 2016. Updated instructions to monitor and adjust the concentration of hydrogen peroxide in the water circuit to limit microbial growth [41]
Safety Notice: 1 December 2016. Risks related to extracorporeal circulation instrumentation [48]
Safety Notice: 14 December 2016. Revised Heating Unit HU35 disinfection procedure [49]
Safety Notice: 16 December 2016. Safety Instructions HCU 40 [50]
Safety Notice: 29 October 2018. Updated instructions to monitor and adjust the concentration of hydrogen peroxide in the water circuit to limit microbial growth [51]
